# Subtypes of stuttering determined by latent class analysis in two Swiss epidemiological surveys

**DOI:** 10.1371/journal.pone.0198450

**Published:** 2018-08-07

**Authors:** Vladeta Ajdacic-Gross, Laura Bechtiger, Stephanie Rodgers, Mario Müller, Wolfram Kawohl, Roland von Känel, Margot Mutsch, Wulf Rössler, Erich Seifritz, Enrique Castelao, Marie-Pierre F. Strippoli, Caroline Vandeleur, Martin Preisig, Peter Howell

**Affiliations:** 1 Department of Psychiatry, Psychotherapy and Psychosomatics, Psychiatric Hospital, University of Zurich, Zurich, Switzerland; 2 ZInEP, The Zurich Program for Sustainable Development of Mental Health Services, University of Zurich, Zurich, Switzerland; 3 Epidemiology, Biostatistics and Prevention Institute, University of Zurich, Zurich, Switzerland; 4 Department of Consultation-Liaison Psychiatry and Psychosomatic Medicine, University Hospital Zurich, Zurich, Switzerland; 5 Collegium Helveticum, University of Zurich and Swiss Federal Institute of Technology, Zurich, Switzerland; 6 Institute of Psychiatry, Laboratory of Neuroscience (LIM27), University of Sao Paulo, Sao Paulo, Brazil; 7 Department of Psychiatry, Lausanne University Hospital, Prilly, Switzerland; 8 Department of Experimental Psychology, University College, London, United Kingdom; Georg-August-Universitat Gottingen, GERMANY

## Abstract

**Aims:**

Associations between stuttering in childhood and a broad spectrum of risk factors, associated factors and comorbidities were examined in two large epidemiological studies. Subtypes of stuttering were then identified based on latent class analysis (LCA).

**Methods:**

Data were from two representative Swiss population samples: PsyCoLaus (N = 4,874, age 35–82 years) and the ZInEP Epidemiology Survey (N = 1,500, age 20–41 years). Associations between stuttering and sociodemographic characteristics, familial aggregation, comorbidity and psychosocial risk / associated factors were investigated in both samples. LCAs were conducted on selected items from people in both samples who reported having stuttered in childhood.

**Results:**

Initial analyses linked early anxiety disorders, such as separation anxiety disorder and overanxious disorder, to stuttering (PsyCoLaus). ADHD was associated with stuttering in both datasets. In the analyses of risk / associated factors, dysfunctional parental relationships, inter-parental violence and further childhood adversities were mutual predictors of stuttering. Moreover, comorbidities were seen with hay fever, asthma, eczema and psoriasis (PsyCoLaus). Subsequent LCA identified an unspecific group of persons who self-reported that they stuttered and a group defined by associations with psychosocial adversities (ZINEP, PsyCoLaus) and atopic diseases (PsyCoLaus).

**Conclusions:**

The two subtypes of developmental stuttering have different risk / associated factors and comorbidity patterns. Most of the factors are associated with vulnerability mechanisms that occur early in life and that have also been linked with other neurodevelopmental disorders. Both psychosocial and biological factors appear to be involved in the etiopathogenesis of stuttering.

## Introduction

Stuttering is a common neurodevelopmental disorder which in most cases starts before four years of age and has a lifetime prevalence of up to 8.5% [1]. Remission occurs before teenage in about 75% of cases [2]. Studies designed to identify risk and associated factors for stuttering have examined child samples predominantly [3, 4] because this age-group has a higher chance of being affected than is the case with adult samples [5]. Research into stuttering epidemiology has two important drawbacks: 1) Comprehensive information about risk / associated factors and comorbid conditions obtained from adults and from population studies [6–8] is sparse. However, many potential vulnerabilities are only noticeable post childhood. 2) Epidemiological information has not been used in subtyping of stuttering, even though it has been used successfully to subtype other neurodevelopmental and early-onset neuropsychiatric disorders [9–12]. Clinical, linguistic and neurophysiological investigations are the predominant approaches in subtyping of stuttering [13–17]. Information about the heterogeneity of stuttering subtypes, both in terms of different outcomes and in terms of etiopathogenetic pathways, is only become available recently. For example, Neumann and colleagues [2017 #275] proposed subtypes involving two forms of acquired (psychogenic and neurogenic) and two forms of developmental (syndromal and the non-syndromal) stuttering subtypes. The non-syndromal develops in childhood without a detectable cause [18] and constitutes the commonest subtype of stuttering. This subtype needs alternative and detailed examination.

Gender (being male) is the dominant risk factor for stuttering, as also applies to other neurodevelopmental disorders. Examples include attention deficit hyperactive disorder (ADHD) [19], conduct disorder [20], tics and the Gilles de la Tourette syndrome (GTS) [9, 21]. These neurodevelopmental disorders are the second-most prominent set of comorbidities with stuttering. The male to female ratio in adults who stutter is estimated at around 4:1 and this gender ratio is higher in those who persist, than in those who recover, from stuttering [5, 22]. Familial aggregation is common [1] but males predominate even when there is no familial predisposition [23]. Neuropsychiatric conditions such as parental anxiety and parental obsessive-compulsive disorder (OCD) are associated with stuttering [8]. Stuttering is also associated with delays in speech development, other speech problems, and dyslexia [24, 25]. Additional risk / associated factors where the evidence is more limited include socioeconomic status, prenatal /perinatal / postnatal brain damage, brain damage related to maternal alcohol consumption during pregnancy, low birth weight, head trauma and head injury, low intelligence, impaired child-parent interactions, conflicts with parents, negative parental reactions to normal childhood dysfluency, emotional reactivity and a sensitive temperament [8, 16, 26–32]. The most frequently investigated comorbid condition with stuttering is anxiety and, more particularly, social anxiety [33–39]. Another common comorbidity of stuttering is ADHD [8, 24, 30, 39, 40].

An alternative perspective about risk factors and comorbidities emerges from the PANDAS model (Pediatric Autoimmune Neuropsychiatric Disorders Associated with Streptococcal infections) [41]. PANDAS focuses on infections caused by group A streptococci during the first few years of life. An autoimmune influence due to autoantibodies directed against basal ganglia tissue is postulated. PANDAS applies to neurodevelopmental disorders such as GTS and ADHD [42, 43] and a link with stuttering has been reported in a case study [44]. In addition to streptococci, several other infectious agents have been investigated as causative factors in mental disorders, primarily in psychosis [45, 46] and anxiety disorders [47]. These agents merit more consideration with respect to stuttering, as they may play a causative role in this condition too.

In the following article, two studies are reported that were designed to shed light on the spectrum of risk / associated factors in stuttering, including comorbidities across psychiatric and somatic diseases. The two studies examined data from two large Swiss population samples—the ZInEP and the PsyCoLaus epidemiological surveys. The samples were obtained in a psychiatric context. Both samples included middle-aged adults. Hence they provided a comprehensive picture of associated conditions and comorbid disorders. The ZInEP study obtained a large range of psychosocial variables and adversities; the PsyCoLaus study extended this to a wide spectrum of somatic conditions, parameters and diseases.

The aim of the present study was to determine subtypes of stuttering in childhood based on risk / associated factors reported by participants in the two extensive and detailed epidemiological surveys. The analysis strategy followed the common two-step design of investigating bivariate and multivariate analyses. Preliminary selection of associated variables was based on previous reports about stuttering and its association patterns with ADHD and other neurodevelopmental disorders. In the multivariate step, the data were analysed using latent class analysis (LCA), which is a person-centered statistical analysis model, suitable for determining subgroups of persons who stutter/ed (PWS).

## Study 1: The ZInEP epidemiology survey and stuttering: Psychosocial risk / associated factors, familial aggregation and psychiatric comorbidities

### Study 1: Data and methods

#### The ZInEP epidemiology survey: Design, sample, main instrument

The ZInEP epidemiology survey is a sub-project of the Zurich Program for Sustainable Development of Mental Health Services (ZInEP is the German acronym for Zürcher Impulsprogramm zur nachhaltigen Entwicklung in der Psychiatrie) [48]. This epidemiological survey comprises four parts: 1) a brief telephone screening, 2) a comprehensive semi-structured interview supplemented by self-report questionnaires, 3) a battery of social and neurophysiological tests that focus on stress and psychotic symptoms, and 4) a longitudinal survey component. The survey was carried out between August 2010 and September 2012 (see Table 1 for details). For the current article, data from the first two parts of the ZInEP study were used.

**Table 1:** Characteristics of PsyCoLaus and the ZInEP epidemiology survey.

	PsyCoLaus 1st survey	PsyCoLaus 2nd survey	ZInEP
year of interview(s)	2004–7	2009–12	2010–12
N screened	-		9,829
N sample (baseline interviews)	3,720	1,154	1,500
N after reversing stratification	-		3,600
age range	35–66	42–82	20–41

First, a representative sample of 9,829 participants who lived in the canton of Zurich and were aged between 20 and 41 years at the commencement of the study, were screened using a Computer Assisted Telephone Interview (CATI) format for Symptom Checklist-27 (SCL-27) [49]. The participants who were screened were selected at random from the communal public authority register. The response rate for participants who were reached by telephone was 73.9% with an overall response rate of 53.6%.

Once screening was completed, 1,500 participants were selected based on a stratified sampling procedure. This selected at random 60% of the high-scorers and 40% of the low-scorers (the cut-off criterion being the 75^th^ percentile of the Global Severity Score of the SCL-27 [49]). Of the participants who were invited, 64.9% attended the interview. They were interviewed using a shortened version of the SPIKE (Structured Psychopathological Interview and Rating of the Social Consequences of Psychological Disturbances for Epidemiology), which was developed by Jules Angst in the Zurich Study (for more details see [50, 51]). Diagnoses of common mental disorders (CMD) were computed as 12-month prevalence rates based on Diagnostic Statistical Manual IV (DSM-IV) criteria. Exceptions included diagnoses of:

- generalized anxiety disorder (GAD). Here a modified time criterion of one month was used;
- agoraphobia, where the criteria were modified according to DSM-V (number of symptoms, coding of impairment). This was done because the DSM-IV criteria for agoraphobia diverged from the common diagnosis scheme;
- neurasthenia, which was based on ICD-10 criteria;
- mania / hypomania and bipolar disorder, which were adapted according to the criteria of the Bridge Study [52];
- ADHD was assessed by the Wender Utah Rating Scale (Short Form), yielding a dimensional rather than a categorical diagnosis [53].

#### Stuttering

The questions about stuttering followed immediately after questions about dyslexia and other language impairments. The probe question for stuttering was: “Did you stutter in childhood or adolescence?” When the answer was affirmative, more detailed information was sought. Specifically, based on the community study of Craig and colleagues [54], participants were asked about the following stuttering symptoms: a) blocks whilst speaking words; b) repetition of words/phrases; and c) difficulties with specific sounds or syllables as in part-word repetitions and prolongation. The participants who confirmed that their speech impairment evidenced at least one of these symptoms were considered to be PWS. As further selection criteria, stuttering had to have lasted for at least three months [54] with onset before age 12. Age 12 was chosen because it is often used in studies that look at various aspects of brain and social development and also as a cutoff to separate developmental from adult onset of stuttering. These criteria resulted in three operational definitions: self-reported stuttering; stuttering specified by any of the three symptoms; and the latter plus the criterion that stuttering lasted for at least three months. The defined groups were compared to check for any redundancy by considering their pattern of associations with further variables.

#### Risk / associated factors and socioeconomic variables

The familial aggregation of substance abuse and mental problems was assessed in first-degree relatives. With respect to stuttering, aggregation for both the first-degree relatives and the extended family (grandparents, cousins, aunts, uncles) was conducted. Childhood adversities were assessed by criteria adapted from the Zurich Study [55] and the Childhood Trauma Questionnaire (CTQ) [56]. Obstetric complications were assessed with questions based on the Obstetric Complications Scale [57]. The education level was represented by three levels (operationally defined as: 1) *low*: basic school and apprenticeship level; 2) *medium*: pre-university and high-level technical schools; 3) *high*: university) [48].

#### Ethics approval

The ZInEP study was approved by the Ethics Committee of the Canton of Zurich (KEK), and is in accordance with the declaration of Helsinki of the World Medical Association.

#### Statistical analysis

Descriptive models, cross-tabulations and regression models were estimated for basic statistical analysis. Programming and the bulk of the analyses were carried out using SPSS Statistics (Version 23). Adjustment using appropriate weights and corrected confidence intervals (CI) to account for the sample stratification was performed in ZInEP by SAS survey procedures SURVEYFREQ, SURVEYMEANS and SURVEYLOGISTIC (Version 9.4).

Variables with low cell frequencies (<= 5) in crosstabulation with stuttering were omitted from further analyses. Variables with at least trend level significance are reported since some cell frequencies were still low. In the multivariate analysis, LCA was employed. LCA is a classification model like factor analysis (FA) or cluster analysis (CA). In contrast to FA, which is a variable-centered approach that places variables along dimensions or factors, LCA and CA are person-centered approaches, i.e., they aim to group individuals into homogeneous classes or clusters [58, 59]. In LCA, the proportion of participants in each class is determined by class probabilities, whereas the item-response probabilities represent the probabilities related to positive responses on each variable or item category.

Depending on the selection of variables, the classes in the LCA can be interpreted as representing subtypes of a disorder or disease. LCAs often establish subgroups that differ in quantitative terms, i.e. represent more and less severely affected subtypes. LCAs can also be used to determine subgroups that differ qualitatively and thus provide indications about different etiopathogenetic mechanisms.

LCA was conducted using Mplus version 7 [60] and Latent Gold version 4.5 to validate results [61]. Variables representing early exogenous risk / associated factors, e.g., family related childhood adversities, and gender (as covariate) were used to conduct the LCAs whereas school / peer-related adversities, comorbid conditions and familial aggregation variables were introduced as inactive variables, i.e., were assigned post hoc to the given classes. Models with one through three latent classes were fitted to the data. The LCA models were compared by the Akaike information criterion (AIC) and the Bayesian information criterion (BIC) fit indices to guide model selection [62]. Lower values of these information criteria suggest a better model fit. The AIC tends to overestimate, and the BIC tends to underestimate the number of classes [63].

### Study 1: Results

Of the 1,488 people in the ZInEP sample who responded to the probe question about language and speaking, *n* = 81 reported having stuttered before they reached the age of 12 years. This gives a prevalence of 5.4% (CI 3.4–7.5) after weighting to account for the sample stratification. When additional criteria were applied in order to restrict the sample further, only *n* = 57 (3.7%) reported having at least one of the three specific stuttering criteria of blocks, repetition of words/phrases, struggles with specific sounds or syllables. Blocks (*n* = 51) and struggling (*n* = 41) were notably more common than the repetition symptom (*n* = 11). Thirty-four participants reported having had one symptom, 22 participants reported having had two symptoms and one participant reported having had all three symptoms. Forty-nine (3.2%) of the participants had symptoms that lasted for at least three months (Table 2). The mean age at onset was 6.0 (CI 5.3–6.7) years for retrospectively reported stuttering. Men predominated, with a ratio of 5:1 in self-labelled stuttering and approximately 6:1 when more restrictive definitions were applied. Additionally, neither education nor any obstetric variable showed any significant association with stuttering (results not shown).

**Table 2:** ZInEP: Frequencies and prevalence rates of stuttering according to different definitions.

stuttering items / definitions	N	rate (weighted, CIs)
did you stutter in childhood or adolescence?	85	5.5 (4.1–6.9)
did you stutter, onset by age of 12	81	5.4 (4.0–6.8)
frequency of specific symptoms (onset by age of 12)		
a) blocks while speaking words	51	3.2 (2.1–4.2)
b) repetition of words/phrases	11	0.8 (0.2–1.4)
c) struggling with specific sounds or syllables / prolongation of speech /	41	2.4 (1.5–3.3)
stuttering according broad definition (either symptom a or b or c)	57	3.7 (2.6–4.9)
stuttering according narrow definition (either symptom a or c)	55	3.6 (2.4–4.7)
stuttering according broad definition plus min. of 3 months’ duration	49	3.2 (2.1–4.3)

Familial aggregation of stuttering (Table 3) was a strong predictor of stuttering with odds ratios (ORs) > 5 using any of the stuttering definitions. However, this association was limited to first-degree relatives. Furthermore, parents’ mental health as reported by study participants was indicated by reports of panic attacks, manic behaviour and drug, but not alcohol abuse.

**Table 3:** ZInEP: Familial aggregation and psychosocial risk / associated factors related to stuttering. Odds ratios and confidence intervals (95%), adjusted for sex.

	stuttering probe question	stuttering, any of 3 symptoms	stuttering, any of 3 symptoms, 3 months
familial aggregation			
stuttering (nuclear family)	5.5 (2.4–12.7)	6.4 (2.5–16.6)	7.7 (3.0–20.2)
stuttering (extended family)	1.4 (0.5–3.5)	1.3 (0.4–3.8)	0.7 (0.2–1.9)
panic attacks (nuclear family)	2.2 (1.1–4.7)	3.0 (1.3–6.9)	2.1 (0.8–5.4)
mania, manic behaviour (nuclear family)	2.4 (1.1–5.4)	2.9 (1.2–7.0)	2.6 (1.0–6.6)
alcohol abuse / dependence (nuclear family)	0.8 (0.4–1.6)	0.7 (0.3–1.5)	0.6 (0.3–1.6)
medical drug abuse / dependence (nuclear family)	2.2 (0.8–5.5)	3.4 (1.3–9.1)	3.6 (1.2–10.3)
childhood adversities			
felt emotionally abused ^1^	2.1 (1.1–4.2)	2.9 (1.3–6.9)	2.5 (1.0–6.0)
did not live with both parents up to age of 16	1.9 (0.9–4.2)	3.7 (1.6–8.7)	4.6 (1.9–11.1)
parents quarrelled often	1.9 (1.0–3.7)	3.2 (1.4–7.0)	3.2 (1.4–7.3)
felt happy and safe at home	0.4 (0.2–1.0)	0.4 (0.1–1.0)	0.3 (0.1–0.9)
often felt bored at home	3.2 (1.4–7.2)	4.3 (1.7–11.3)	5.4 (2.0–14.2)
liked going to school	0.6 (0.3–1.1)	0.5 (0.2–1.2)	0.4 (0.2–1.0)
frequent fights with peers	2.2 (1.0–4.9)	2.7 (1.0–7.0)	3.4 (1.3–9.0)
frequent quarrels with peers	2.0 (0.9–4.5)	2.5 (1.0–6.4)	2.2 (0.8–6.1)

Notes:

^1^ CTQ (Childhood Trauma Questionnaire) item, dichotomized.

About 30% of the ZInEP participants who stuttered in childhood did not live with both parents (compared to 15% of participants who did not stutter). This association was more pronounced when more restrictive stuttering definitions were used. PWS reported specific childhood adversities, indicating difficult circumstances at home more frequently than participants who did not stutter. This applied to quarrelling parents (ORs between 1.9 and 3.2) and a lack of support in the family ("felt happy and safe at home" with ORs between 0.3 and 0.4; "often felt bored at home" with ORs between 3.2 and 5.4). These adversities appeared to be different from traumatic experiences since, except for a specific emotional abuse item, none of the Childhood Trauma Questionnaire (CTQ) subscales (emotional / physical neglect, emotional / physical / sexual abuse) yielded significant results. PWS were also more frequently involved in conflicts with peers than were non-stuttering respondents (ORs between 2.2 and 3.4).

Associations with mental disorders are listed in Table 4. PWS showed an increased risk for ADHD as assessed by the Wender Utah Rating Scale. Two of the three stuttering variables were associated with hypomania. No association with social phobia was found, even after a subgroup with symptom onset in childhood was differentiated.

**Table 4:** ZInEP: Associations between stuttering and common mental disorders. Odds ratios and confidence intervals (95%), adjusted for sex.

	stuttering probe question	stuttering, any of 3 symptoms	stuttering, any of 3 symptoms, 3 months
social phobia	0.8 (0.3–1.7)	0.8 (0.3–2.1)	1.0 (0.4–2.5)
specific phobia	1.3 (0.5–2.9)	1.4 (0.5–3.7)	1.7 (0.6–4.5)
GAD	1.1 (0.3–3.8)	1.5 (0.4–5.7)	0.6 (0.2–2.2)
agoraphobia	0.7 (0.2–3.2)	1.1 (0.3–4.9)	1.3 (0.3–5.7)
panic disorder	0.9 (0.1–7.0)	1.4 (0.2–10.7)	1.7 (0.2–12.5)
OCD	2.2 (0.8–5.8)	3.0 (1.0–8.8)	2.5 (0.7–8.4)
major depression	0.8 (0.4–1.4)	0.9 (0.9–1.7)	1.0 (0.5–2.1)
bipolar disorder	1.8 (0.5–6.4)	2.8 (0.8–9.9)	3.2 (0.9–11.6)
hypomania	2.9 (1.1–7.1)	3.4 (1.2–9.8)	2.5 (0.8–7.9)
dysthymia	0.9 (0.2–4.2)	1.4 (0.3–6.3)	1.6 (0.4–7.4)
neurasthenia	1.7 (0.5–5.0)	2.4 (0.7–7.8)	1.6 (0.4–6.2)
alcohol abuse / dependence	1.3 (0.4–3.2)	1.1 (0.3–4.3)	1.3 (0.3–5.2)
ADHD (WURS-SF)	1.03 (1.00–1.05)	1.03 (1.00–1.06)	1.04 (1.01–1.07)

Abbreviations:

ADHD, attention deficit hyperactivity disorder; OCD, obsessive-compulsive disorder; GAD, generalized anxiety disorder; WURS-SF, Wender Utah Rating Scale Short Form

**Fig 1:**
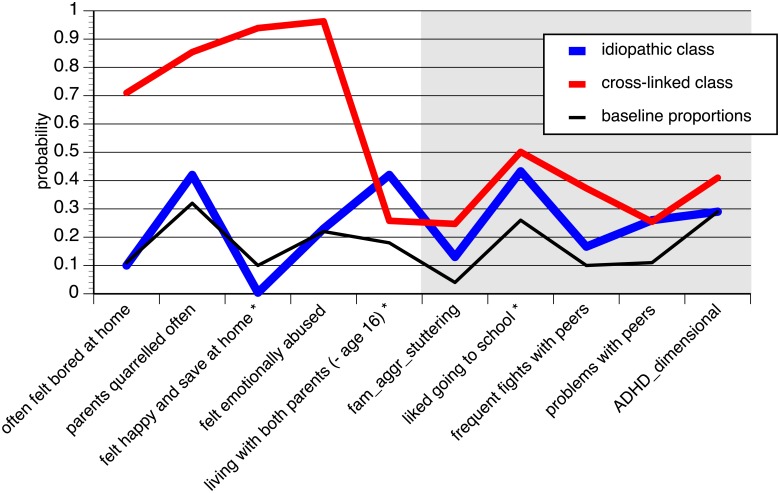
Probabilities of the two class LCA of stuttering and baseline proportions of risk / associated factors in the ZInEP study. Items with asterisks were reversed. The grey area denotes items whose probabilities were assigned post hoc to the latent classes. The items were grouped and their probabilities were connected by lines in order to facilitate examination of the LCA.

In LCA, the fit indices indicated that both the AIC and the BIC were lower (preferable) for the two-class model (AIC: 224, BIC: 244) compared to the one-class model (AIC: 250, BIC: 258). The larger latent class comprised 79% of PWS. Its pattern (see Fig 1, idiopathic class) mostly followed the pattern of the entire sample apart from the dysfunctional parental relationships / broken home item. Familial aggregation of stuttering was more frequently endorsed by this group than by participants who did not stutter, but was reported less frequently than with the second LCA group. This also applied to physical fights and problems with peers. About 75% of the PWS in the first LCA class were men.

For the second latent class (21% of PWS, cross-linked class), values for all the selected psychosocial adversities increased except for the broken home variable (see above). PWS in this group displayed a higher percentage of familial aggregation of stuttering, more problems in school and with peers and also higher scores on ADHD scales. The sex ratio was equal (50% men, 50% women).

## Study 2: The PsyCoLaus study and stuttering: Familial aggregation, psychiatric and somatic comorbidities

### Study 2: Data and methods

#### The PsyCoLaus sample and instruments

The PsyCoLaus study [64] is the psychiatric part of the population-based CoLaus study [65]. CoLaus / PsyCoLaus together constitute a cohort study conducted in the city of Lausanne (Switzerland). Initial assessments were made 2003–6, and follow-ups were conducted in 2009–12 and in 2014–17. The main focus of CoLaus is on cardiovascular and other somatic diseases, whereas the major aim of the PsyCoLaus part is to record data on the prevalence of psychiatric syndromes.

The initial 2003 CoLaus sample consisted of a representative group of n = 6,734 inhabitants of Lausanne aged 35–75 years. The participants were assessed at an outpatient clinic [65, 66]. The study protocol involved the collection of clinical data and blood samples, and a semi-structured questionnaire-based interview. One year later, all participants in CoLaus, aged between 35 and 66 years (n = 5535), were invited to participate in PsyCoLaus; a total of n = 3,720 individuals (67%) agreed to take part [64].

At the first somatic follow-up assessment (CoLaus), diagnostic information was obtained from 5228 (78%) of the n = 6734 participants who had participated at baseline. Similarly, the participation of those with both somatic and psychiatric baseline evaluations was high at 87% (3,188 out of the 3,673 participants in PsyCoLaus who were still alive). In addition, participants who had missed the psychiatric part of the baseline examination (including those above 66 years at initial assessment) were asked to complete it after the first CoLaus follow-up, thus extending the age range to 82 years; 1,154 of the participants completed this. Therefore, the total sample comprising baseline data from both interviews summed to N = 4,874 (Table 1).

The psychiatric part of the assessment within the PsyCoLaus study included the French version of the semi-structured Diagnostic Interview for Genetic Studies (DIGS) [67, 68]. The DIGS collects information on a broad spectrum of DSM-IV Axis I criteria and, moreover, on the course and chronology of comorbid features [64]. The brief phobia section of the DIGS was replaced by the corresponding, but more extensive, sections of the Schedule for Affective Disorders and Schizophrenia—Lifetime and Anxiety disorder Version (SADS-LA) [69]. This elicited detailed information on DSM-IV criteria for agoraphobia with or without panic attacks, social phobia and specific phobias. The DIGS and the SADS-LA statistics represent lifetime diagnoses of CMD. High inter-rater and test-retest reliability of the French version of the DIGS have been established for major mood and psychotic disorders [67] as well as for substance use and antisocial personality disorders [70]. Similarly, for the anxiety sections of the French version of the SADS-LA, inter-rater and test-retest reliability are good [71].

The following question was asked (in French) to assess stuttering: “Did you have problems with stuttering during your childhood?” The permitted answers were: “Yes”, “No” or “I don’t know”. If the answer was “Yes”, participants were asked at what age the stuttering had first appeared and if and when it had disappeared. Cases where onset of stuttering was reported to have occurred after the age of 12 were excluded from the analysis.

#### Ethics approval

The PsyCoLaus study was approved by the ethics committee of the University of Lausanne [64]. All participants gave their written informed consent. The study is in accordance with the declaration of Helsinki of the World Medical Association.

#### Familial aggregation

The familial aggregation of mental and substance use problems were assessed by the semi-structured Family History—Research Diagnostic Criteria (FH-RDC) [72, 73]. The information was aggregated into groups of mental disorders comprising: neurodevelopmental disorders (those that typically start during childhood: tic disorders, ADHD, conduct disorder, oppositional defiant disorder); early-onset anxiety disorders (those that typically start during childhood: separation anxiety disorder, overanxious disorder, specific phobias, social phobia); late-onset anxiety disorders (those that typically start after adolescence: generalized anxiety disorder, panic, agoraphobia); mood disorders (those that typically start after adolescence: major depressive disorder, dysthymia, bipolar disorder); and substance use disorders (those that typically start during or after adolescence: alcohol, cannabis, other illicit drug abuse / dependence). Further disorders that were covered by the FH-RDC were obsessive compulsive disorder and schizophrenia.

#### Further risk / associated factors, somatic and infectious diseases

Information about risk / associated factors, childhood adversities, infectious diseases and other related conditions was obtained by self-report with an extended version of the medical history part of the DIGS (self-reported). Participants were asked whether they had ever been diagnosed with a variety of infectious diseases, diseases of the nervous system, cardiovascular, respiratory, gastrointestinal, metabolic and dermatological conditions, as well as allergies and hormonal problems. For each disease group, a screening question was asked and followed up in cases where there was an affirmative response.

#### Statistical analysis

Basic descriptive models and bivariate analyses adjusted for sex were applied to assess the associations between stuttering and variables of interest by obtaining ORs and 95% CI. The significance level was set at p < 0.05 and trend level was p < 0.1. All analyses and programming were carried out using SPSS Statistics version 22. For details of LCA, see the description in Study 1. As was the case in the ZInEP analysis, only variables representing early exogenous risk / associated factors were used to conduct the LCAs (in PsyCoLaus: atopic diseases, inflammatory diseases with a systemic background, family related childhood adversities). Further adversities, comorbid conditions and familial aggregation markers were introduced as inactive variables, i.e., were assigned post hoc to the given classes. As was the case in the ZInEP analysis, variables with at least trend level significance were included in the LCA, whereas variables with low cell frequencies (< = 5) in crosstabulation with stuttering were omitted irrespective of te significant results in bivariate analysis.

### Study 2: Results

Out of the 4,874 participants in the PsyCoLaus sample who responded to the question about stuttering, 118 reported having stuttered as a child (i.e. before the age of 12). The prevalence in the sample was 2.4%. Of these, 71 (60%) were male and 47 (40%) were female. This yielded a male-to-female sex-ratio slightly below 2. The mean age at stuttering onset was retrospectively reported as 5.8 years (CI 5.4–6.2).

Familial aggregation of mental disorders (Table 5) emerged with respect to neurodevelopmental disorders and early anxiety disorders. Stuttering was not associated with education level (results not shown). However, participants who stuttered in childhood suffered more frequently than other participants from learning problems and dyslexia. Childhood adversities, such as not living with both parents beyond the age of 16, fights among parents and fear of parental maltreatment were identified in bivariate analyses as further potential risk factors for stuttering.

**Table 5:** PsyCoLaus: Familial aggregation of mental disorders and psychosocial factors related to stuttering. Odds ratios and confidence intervals, adjusted for sex (95%).

	OR (CI)
familial aggregation	
neurodevelopmental disorders	2.0 (1.2–3.4)
early anxiety disorders	1.7 (1.1–2.6)
late anxiety disorders	1.1 (0.6–2.1)
mood disorders	1.4 (0.9–2.1)
substance use disorders	1.6 (0.9–2.8)
learning problems in childhood	4.4 (2.8–7.0)
reading problems in childhood	5.0 (3.1–8.2)
fear of parental maltreatment	2.9 (1.8–4.5)
fights among parents	1.7 (1.1–2.8)
did not live with both parents up to age of 16 (broken home)	1.8 (1.2–2.6)

The analysis of neurodevelopmental and psychiatric comorbidities of stuttering in PsyCoLaus (Table 6) revealed associations with neurodevelopmental disorders such as ADHD and, at trend levels, conduct disorder and tics / GTS with ORs around 2.5. Among anxiety disorders, separation anxiety disorder, overanxious disorder and early onset social phobia were associated with stuttering (ORs between 2 and 3.5).

**Table 6:** PsyCoLaus: Associations between stuttering and neurodevelopmental / common mental disorders. Odds ratios and confidence intervals (95%), adjusted for sex.

	OR (CI)
ADHD	2.4 (1.0–5.5)
Tourette syndrome	2.2 (0.9–5.5)
conduct disorder	2.2 (1.0–4.7)
oppositional defiant disorder	0.9 (0.2–3.6)
separation anxiety disorder	2.3 (1.2–4.4)
overanxious disorder	3.5 (2.1–5.8)
specific phobia	1.5 (0.9–2.4)
social phobia	1.5 (0.9–2.5)
social phobia / early onset of symptoms^1^	1.8 (1.0–3.2)
social phobia / late onset of symptoms^2^	0.6 (0.2–2.5)
GAD	1.7 (0.6–4.8)
agoraphobia	1.6 (0.7–3.7)
panic disorder	1.0 (0.4–2.7)
PTSD	1.3 (0.5–3.3)
OCD	2.3 (0.7–7.6)
MDD	1.2 (0.8–1.8)
bipolar disorder	0.5 (0.1–3.6)
dysthymia	0.5 (0.1–2.1)
bulimia	0.9 (0.1–6.7)
alcohol abuse / dependence	1.4 (0.8–2.3)

Abbreviations:

ADHD, attention deficit hyperactivity disorder; OCD, obsessive-compulsive disorder; MDD, major depression disorder; GAD, generalized anxiety disorder; PTSD, posttraumatic stress disorder.

Notes:

^1^ until age 8.

^2^ age 9 or later.

None of the most common childhood infections such as pertussis, chickenpox, measles, mumps and rubella showed a noteworthy association with stuttering. Associations with streptococcal infections were unclear because frequency of cases was low. Among atopic and skin diseases, hay fever had the strongest association (OR 2.4, CI 1.6–3.5), followed by asthma, eczema and psoriasis, all with ORs around 2 (Table 7). Finally, urticaria and acne were not associated with stuttering.

**Table 7:** PsyCoLaus: Associations between stuttering and skin / atopic diseases. Odds ratios and confidence intervals (95%), adjusted for sex.

	OR (CI)
hay fever	2.4 (1.6–3.5)
asthma (allergy)	2.1 (1.2–3.9)
eczema (allergy)	2.4 (1.3–4.5)
urticaria	1.6 (0.7–3.6)
acne	0.6 (0.3–1.3)
psoriasis	2.0 (1.0–3.8)

**Fig 2:**
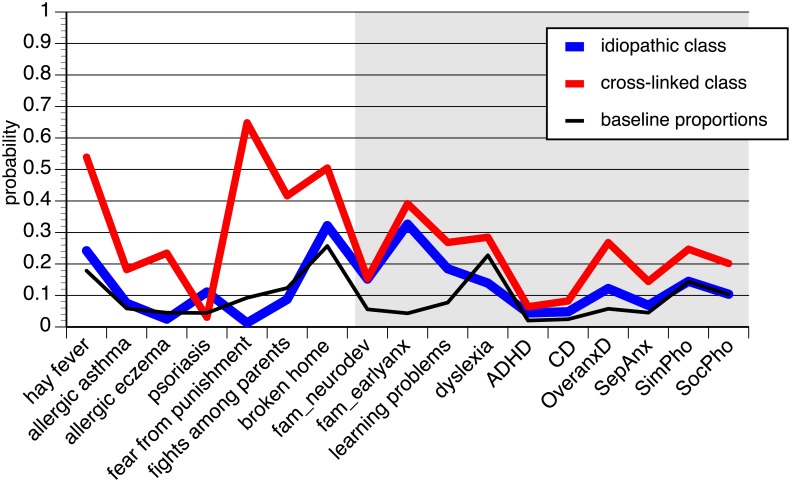
Probabilities of the two class LCA of stuttering and baseline proportions of risk / associated factors in the PsyCoLaus study. The grey area denotes items whose probabilities were assigned post hoc to the latent classes. The items were grouped and their probabilities were connected by lines in order to facilitate examination of the LCA.

The LCA fit indices for the one- and the two-class models were similar (AIC: 786 vs. 769; BIC: 805 vs. 813), the AIC being slightly lower, and the BIC slightly higher in the two-class model. The two-class solution corresponded with the outcome of the LCA using the ZInEP data. The proportions in the classes were comparable to the LCA with ZInEP data: 67% vs. 33%. Again, the pattern of the larger latent class mostly followed the pattern in the rest of the sample (see Fig 2). Exceptions were: familial aggregation with similar probabilities in both classes and psoriasis with marked results. Intermediate values occurred for hay fever, broken home, and, assigned post hoc to the class, familial aggregation of early anxiety disorders, learning problems, ADHD, CD, overanxious disorder. Males predominated in this class (70%). Class 2 had increased values for both the somatic and the psychosocial risk / associated factors (except psoriasis, see above). Post hoc assignment of further variables showed that PWS from this group had similar values for familial aggregation variables, and higher values for learning problems, dyslexia and comorbid neurodevelopmental and early anxiety disorders than PWS from class 1. In class 2, there were slightly more females than males (60%).

## Discussion

These are the first studies that identify stuttering subtypes based on a broad spectrum of risk / associated factors and comorbidities derived from LCA models. The data employed were taken from two large Swiss epidemiological surveys and the analysis design allowed a replication of findings across the studies. The ZInEP Epidemiology Survey provided insights into associations with psychosocial factors, and comorbidities with common mental disorders (Study 1), whereas the PsyCoLaus data allowed associations with infectious and somatic diseases and comorbidities with neurodevelopmental disorders to be investigated (Study 2). The results supported the notions that different subtypes of stuttering in childhood exist and that different etiopathogenetic pathways are involved in stuttering. This is a constellation which markedly, and unsurprisingly, resembles other neurodevelopmental and neuropsychiatric disorders [74].

### Specific results

The LCAs yielded similar two-class solutions in both studies with the smaller class relying on most of the risk / associated factors mentioned above and characterized by a higher impact on comorbidity. In contrast, the larger class displayed a moderate association only with psoriasis and moderate comorbidities with other neurodevelopmental and mental disorders.

Psychosocial risk / associated factors identified in the present data were dominated by broken home and adversities in the nuclear family, including disintegration of the nuclear family, interparental violence, and a lack of support at home. When compared with analyses with other mental disorders, for example obsessive compulsive disorder [75] or specific phobias [76], the range of psychosocial risk / associated factors in stuttering was limited in number and included mainly chronically stressful psychosocial conditions in the family in combination with interparental violence or fear of maltreatment. However, the pattern of psychosocial risk / associated factors found in the two present studies did not confirm parental alcohol abuse or dependence [8], as reported in previous studies. Also, evidence for risk factors such as obstetric complications did not emerge [77].

The analyses involving somatic conditions (PsyCoLaus) indicated that hay fever, other atopic diseases, but also psoriasis, have a greater likelihood of coexisting with stuttering than expected by chance. Notably, an association between atopic diseases and speech disorders has recently been established [78, 79]. Moreover, there is a similar constellation in ADHD including associations with allergic rhinitis, asthma and eczema [80–84]. Another parallel between stuttering and ADHD is in childhood adversities [85], although the significance of this association has been interpreted in different ways in these two research domains. Both atopic diseases and childhood adversities are consistently associated with other early onset disorders such as tics [86, 87] and early anxiety disorders [88, 89]. Based on this, it is difficult to argue that stuttering and ADHD have special co-roles despite their having very similar association and comorbidity patterns.

Stuttering and ADHD not only display a similar constellation of comorbidities but are also directly interlinked [39], which was also shown in both ZInEP and PsyCoLaus samples. Moreover, in PsyCoLaus associations were found with conduct disorder and tics / GTS (at trend level) but also with anxiety disorders typically manifesting in childhood, i.e., separation anxiety disorder, overanxious disorder and early onset social phobia. While comorbidity with social phobia has been the focus of research so far, this study showed that the framework of comorbidities of stuttering entails a larger spectrum of disorders starting in childhood. However, stuttering showed no links to mood and late anxiety disorders except hypomania (in ZInEP). This is particularly remarkable, since the samples in the two studies comprised adults and thus, in contrast to most studies with PWS, also cover the lifetime prevalence of mental disorders. Summarizing, the moderate comorbidity with other disorders is an outstanding and challenging feature of stuttering, as has been noted previously [90].

#### Approaching subtypes of stuttering

Risk / associated factors and comorbidity patterns provide a core approach, among many other possible approaches [76], to subtyping of disorders. Meanwhile, they have rarely been employed in stuttering research [30] despite a long tradition of interest in subtyping [13, 15, 16]. In both Swiss samples analysed in this study, the LCA of risk / associated factors yielded two classes with similar makeup: one class was characterized by psychosocial adversities in the family and atopic diseases and it may be labelled as the cross-linked stuttering class. The other class was characterized by absence of most risk / associated factors and may be labelled as the idiopathic stuttering class. Familial aggregation was important in both classes, whereas comorbidity with ADHD and early anxiety disorders was more pronounced in the cross-linked class. The latter finding is in line with the study of Alm and Risberg [30]. Evidence from epidemiological studies on neurodevelopmental and mental disorders suggests that comorbidity is an indicator of severity; consequently, it might be expected that the cross-linked class represents a more severe subtype of stuttering.

The configuration of risk / associated factors and comorbidities in the cross-linked stuttering class is not surprising given that stuttering is associated with ADHD and early anxiety disorders. All these disorders share risk / associated factors which include both psychosocial adversities and immunological processes [74]. Particularly, the scenarios in ADHD and stuttering look likely to be similar.

The idiopathic stuttering class is more difficult to interpret. The moderate associations with ADHD, CD and overanxious disorder indicate mutual hidden regularities in their etiopathogenesis. The association with psoriasis remains to be replicated. Moreover, the familial aggregation variables indicate an additional role of genetic factors also in this class. However, larger data bases are required to tease out and examine further hypotheses.

#### Future work

The discussion of different subtypes of stuttering provides a basis for ensuing hypotheses and explorations. Basically, we hypothesize that neurodevelopmental disorders and mental disorders with an early onset derive from disturbances of brain development introduced by immunological and endocrine imbalances during the critical early periods of life [91].

There are many parallels between immunological problems and stuttering. Immunological imbalances emerge not only due to infections and atopic diseases, but also due to psychosocial adversities [92–94]. Conversely, inflammatory processes impact neuroendocrine circuits [95, 96]. We hypothesize that the neurophysiological processes related to atopic diseases and psychosocial adversities overlap to a great extent [97, 98] and that they impact stuttering in a similar way to the way they impact ADHD and other neurodevelopmental and mental disorders.

Childhood adversities due to dysfunctional parental relationships and interparental violence do not begin at a specific age of the child but are likely to be present at the beginning of parenthood. They impact brain development both via immunological and endocrine pathways [99]. A similar early impact is apparent in atopic diseases. Neonates and infants display Th2 shifted immune responses which are basically related to atopies [100, 101], notably boys having a distinctly stronger shift than girls resulting among others in higher asthma rates [102]. The shift diminishes rapidly before the age of 2–3 years and at a slower rate thereafter, thus providing the time window for strong Th2 related immune system imbalances. This is the same time window when neurodevelopmental disorders with a male predominance are considered to emerge. Moreover, a strong Th2 shift at the beginning of life is more likely to result in immune system programming [103] which favours atopic diseases later in life. This might also explain why associations with atopic diseases are apparent in this study, even though the latter have a later onset than stuttering.

There are two noteworthy implications. First, assessing the timing and the sequence of vulnerabilities and stuttering onset is critical. There are vulnerabilities related to childhood adversities and atopies (i.e., immune system imbalances related to the Th2 shift) that are readily assessable with prospective studies. Other vulnerabilities such as immune system imbalances and programming are currently better assessed with retrospective studies.

Second, if disturbances of brain development interfere very early in life, they could cause more complex and heterogeneous problems involving more heterogeneous brain networks [104]. In stuttering, the brain networks and regions involved comprise about a dozen components [17, 105–107], among them not least the cerebellum [107, 108]. Moreover white matter differences [109], long range connectivity problems [110] and problems with control networks which monitor coordination, succession and timing of activation [111] are present. Despite stuttering being narrowly-defined as a speech disorder, the underlying neuropathology suggests extensive and complex influences. A similar degree of complexity can also be found in disorders such as autism, ADHD and schizophrenia. Whilst stuttering resembles other neurodevelopmental disorders in this respect, it contrasts with the unexpectedly few genes associated with stuttering that have been identified to date [112–114]. Since evidence from twin studies indicates major impacts of genetics in stuttering [115–117], it seems that genetic research on stuttering is only just beginning. It has addressed distinctly fewer gene loci of interest compared to disorders with a similarly high degree of complexity. For example, the number of such loci in schizophrenia has reached the level of 200 compared to about 20 in stuttering.

#### Limitations

This study used data from two large epidemiological surveys that employed different methodological approaches, instruments and question formats. The risk / associated factors and the comorbidities were assessed differently across the samples, with some risk / associated factors and comorbidities were only assessed in one of the studies. Notably, ZInEP did not include early anxiety disorders, neurodevelopmental disorders (except ADHD), infectious diseases and detailed somatic conditions. In contrast, PsyCoLaus comprised only a limited number of variables covering psychosocial adversities.

The studies took place in different regional contexts (German and French parts of Switzerland) and involved different generations with different norms and values. Furthermore, the participants in the two samples were adults aged up to 41 years in ZInEP but up to 82 years in PsyCoLaus. This might lead to a recall-bias regarding symptoms and other issues from childhood and youth. This applies not only to stuttering, but also to risk / associated factors and other retrospectively-collected information. However, the information derived from both studies is largely congruent.

An example is the self-reporting of the age of onset of stuttering, which is about 6 years in both studies, that is 2–3 years above the average values reported in clinical studies. Reporting of onset age has been critically discussed in terms of telescoping effects, above all in substance use research [118, 119]. Telescoping implies that the onset age of remote events is shifted towards higher ages. Telescoping would also apply to the psychosocial and somatic risk / associated factors. Therefore, age at onset parameters derived from surveys do not compare to those derived from clinical studies. Nevertheless, there is no evidence that telescoping regarding age at onset varies with respect to all other variables included in the present analyses.

The analysis design relied explicitly on association patterns and on their replication across two different study samples. As usual in pattern recognition analysis (factor analysis, correspondence analysis, LCA etc.), no initial adjustment for multiple testing was carried out [120].

In both samples, the information used was based on self-reports, and stuttering was basically assessed by self-labelling. However, the ZInEP questionnaire included additional criteria that provided more precise stuttering variables. Here the results basically remained unchanged and consistent compared to a simple one question format, therefore suggesting that stuttering had been identified correctly by participants.

Despite their large size, the ZInEP and the PsyCoLaus samples are only moderately suited to analyses of infrequent conditions such as stuttering. The cell-frequencies in cross tabulations often returned small or null cell frequencies. This might also be a reason for the absence of some of the associations reported by other researchers.

## Conclusions

This study suggests that both psychosocial adversities in childhood and biological factors are independently associated with the risk of stuttering. These factors served to determine two classes in LCA, i.e., two subtypes of stuttering, which were replicated in both samples. One subtype, termed the cross-linked class, is associated with factors such as atopic diseases (hay fever, asthma, eczema) and psychosocial adversities in childhood. It is characterized by a range of comorbidities with other neurodevelopmental and early anxiety disorders. The other subtype, the idiopathic class, showed only sporadic associations with other variables (for example, psoriasis) and smoothed associations with few comorbid disorders. Familial aggregation was present in both subtypes.

This is the first study to determine subtypes of stuttering based on a broad range of risk / associated factors and comorbidities using LCA. It appears that stuttering—despite being narrowly defined as a speech disorder—does not substantially differ from most other neurodevelopmental disorders, neither in terms of association patterns nor in terms of heterogeneity nor in terms of complexity of the underlying neuropathology. The results suggest the need for a synthesis of different theories in stuttering research.

## Supporting information

S1 Table Frequencies_table3. Stuttering (any of 3 symptoms) vs. familial aggregation (FA) / childhood adversities (CHAD) / ZInEP data.
(TXT)

S2 Table Frequencies_table3weighted. Stuttering (any of 3 symptoms) vs. familial aggregation (FA) / childhood adversities (CHAD). Weighted frequencies / ZInEP data.
(TXT)

S3 Table Frequencies_table4. Stuttering (any of 3 symptoms) vs. common mental disorders / ZInEP data.
(TXT)

S4 Table Frequencies_table4weighted. Stuttering (any of 3 symptoms) vs. common mental disorders. Weighted frequencies / ZInEP data.
(TXT)

S5 Table Frequencies_table5. Stuttering vs. familial aggregation (FA) / childhood adversities (CHAD) / Psycolaus data.
(TXT)

S6 Table Frequencies_table6. Stuttering vs. common mental disorders / Psycolaus data.
(TXT)

S7 Table Frequencies_table7. Stuttering vs. skin / atopic diseases / Psycolaus data.
(TXT)

S8 Table Frequencies_fig1_for_LCA. Fig 1 / variables used for calculating LCA / ZInEP data.
(TXT)

S9 Table Frequencies_fig2_for_LCA. Fig 2 / variables used for calculating LCA / Psycolaus data.
(TXT)
